# Status epilepticus and cardiopulmonary arrest in a patient with carbon monoxide poisoning with full recovery after using a neuroprotective strategy: a case report

**DOI:** 10.1186/1752-1947-6-421

**Published:** 2012-12-14

**Authors:** Salman Abdulaziz, Ousama Dabbagh, Yaseen Arabi, Suleiman Kojan, Imad Hassan

**Affiliations:** 1 Department of Medicine, King Abdulaziz Medical City, King Fahad National Guard Hospital, PO Box 22490, Riyadh, 11426, Kingdom of Saudi Arabia; 2 Division of Pulmonary, Critical Care and Environmental Medicine, University of Missouri -Columbia, 5 Hospital Drive CE 428, Columbia, MO, 65212, USA; 3 Department of Intensive Care, King Fahad National Guard Hospital, Box 22490, Riyadh, 11426, Kingdom of Saudi Arabia; 4 Department of Neuroscience, King Abdulaziz Medical City, King Fahad National Guard Hospital, Box 22490, Riyadh, 11426, Kingdom of Saudi Arabia

**Keywords:** Cardiac arrest, Carbon monoxide poisoning, Neuroprotective strategy, Seizure, Status epilepticus

## Abstract

**Introduction:**

Carbon monoxide poisoning can be associated with life-threatening complications, including significant and disabling cardiovascular and neurological sequelae.

**Case presentation:**

We report a case of carbon monoxide poisoning in a 25-year-old Saudi woman who presented to our facility with status epilepticus and cardiopulmonary arrest. Her carboxyhemoglobin level was 21.4 percent. She made a full recovery after we utilized a neuroprotective strategy and normobaric oxygen therapy, with no delayed neurological sequelae.

**Conclusions:**

Brain protective modalities are very important for the treatment of complicated cases of carbon monoxide poisoning when they present with neurological toxicities or cardiac arrest. They can be adjunctive to normobaric oxygen therapy when the use of hyperbaric oxygen is not feasible.

## Introduction

Carbon monoxide (CO) poisoning remains one of the most difficult medical emergencies to diagnose because of its non-specific and variable clinical presentation [1]. In Saudi Arabia, accidental (as opposed to deliberate, that is, suicidal) exposure usually results from smoke inhalation from charcoal fires used for heating in rooms that are poorly ventilated during the winter season.

In our report, we describe the presentation and successful management of a woman with significant CO poisoning. We highlight the specific interventions that were employed to safeguard a favorable outcome by using a neuroprotective strategy. We also review the subject in general and advocate a systematic approach to its management based on available local resources. Our patient presented with status epilepticus and cardiopulmonary arrest. She was successfully managed by cerebral protection and normobaric oxygen therapy.

## Case presentation

A 25-year-old Saudi woman with no previous medical illness was brought to the Emergency Department at 8.00 a.m. after being found unresponsive, two hours earlier, at home. She had placed a charcoal fire at her bedside since midnight to warm her room. On arrival to her room, family members found her having brief abnormal generalized body movements and she was unresponsive. There was no history of psychiatric illness, drug misuse, previous suicidal attempts or epilepsy.

On arrival to our Emergency Room, she was comatose with a Glasgow Coma Scale (GCS) score of 3/15. A vital signs examination revealed an oral temperature of 37.0°C, a regular pulse of 114 beats/minute, blood pressure of 127/98mmHg and a respiratory rate of 24 breaths/minute. Her pulse oximetry was 99 percent on room air. Otherwise the results of her general routine examination were unremarkable.

Her serum glucose and routine serum electrolyte levels were normal. Her hemoglobin (Hb) level was 135g/L, and her leukocyte count was 22×10^9^ cells/L with neutrophilia. Her creatinine kinase level was 6489U/L (normal range 38 to 174U/L), creatinine kinase-MB isoenzyme level 143.5U/L (normal range 0.20 to 5.00U/L), aspartate aminotransferase 128U/L (normal range 15 to 40U/L), lactate dehydrogenase 459U/L (normal range 100 to 190U/L) and troponin I was 4.69ng/L (normal range <0.10ng/L). The results of urine toxicology screening tests, including tests for benzodiazepines, cannibinoids, amphetamines, opiates and cocaine, were negative.

Her arterial blood gases on a non-rebreather facial mask at 15L/min oxygen showed pH 7.35, PaCO_2_ 36.2mmHg, PaO_2_ 348.9mmHg, HCO^3-^ 19mmol/L, SaO_2_ 99.8 percent and carboxyhemoglobin (COHb) of 21.4 percent.

An electrocardiogram (ECG) showed sinus tachycardia with no ST segment changes. The results of a brain computed tomography (CT) scan were normal.

The initial impression was of severe carbon monoxide poisoning associated with coma, hypoxic rhabdomyolysis and cardiac muscle injury.

Endotracheal intubation was instituted because of her low GCS. Soon after, she started to have a generalized tonic-clonic seizure that continued for approximately 50 minutes despite frequent intravenous doses of benzodiazepines and intravenous phenytoin loading. Her convulsions finally abated after adding thiopental (100mg intravenously) to the regiment. Soon after that she developed asystole. Sinus rhythm and a recordable blood pressure were re-attained following a 10-minute cardiopulmonary resuscitation. She had not had an electroencephalogram (EEG) during the time of her clinical seizure but a subsequent EEG showed α-coma waves and bilateral sharp epileptiform discharges with no signs of status epilepticus.

In our Intensive Care Unit (ICU), she was mechanically ventilated with FiO_2_ 100 percent and her COHb level normalized after 54 minutes. We used our hospital protocol for severe traumatic brain injury, which is compatible with the Brain Trauma Foundation protocol (2007), in order to achieve brain protection [2]. The protocol included the following: 

(1) management of airway and breathing by early intubation and mechanical ventilation, which was adjusted to maintain a pulse oximetry ≥95 percent and/or PaO_2_ ≥80mmHg and eucapnea with PCO_2_ of 35 to 40mmHg;

(2) maintaining euvolemia using isotonic fluid to keep central venous pressure equal 8 to 10mmHg;

(3) inotropes to maintain mean arterial pressure (MAP) above 80mmHg;

(4) moderate hypothermia (32°C to 34°C);

(5) maintaining normoglycemia (8.1 to 10mmol/L);

(6) intravenous phenobarbitone and phenytoin to prevent seizure recurrence and intravenous fentanyl and midazolam for sedation.

An echocardiogram showed normal left ventricular function and no regional wall abnormalities. A repeat EEG on day seven revealed no evidence of status epilepticus; however, it showed evidence of a moderate degree of encephalopathy that was attributed to multiple factors that would, possibly, include a previous CO poisoning and hypoxic event, on top of the effect of medications used (that is, midazolam, phenytoin and phenobarbitone), since a repeated CT scan of the brain and repeat electrolyte test results were normal.

She remained on mechanical ventilation for 13 days. This was complicated by ventilator-associated pneumonia. She was successfully extubated and started on an intensive rehabilitation program with rapid improvement. She was discharged home fully independent in physical function. A brain magnetic resonance imaging (MRI) scan did not show any signs of CO poisoning three weeks after her initial presentation. A cognitive function assessment was not performed by a psychologist at that time since her mental and psychological functions were recovering significantly. She returned back to work as an office secretary two months after the event with excellent and complete functional recovery and no neurological or psychological deficits. Our patient cordially agreed to attend our clinical meeting discussing her case presentation and management. She confirmed to the audience that she felt both physically and cognitively back to her normal self. Subsequently, she actively participated in an awareness campaign for CO poisoning. Currently, she is a postgraduate student studying abroad.

## Discussion

CO is a non-irritant, colorless, odorless and tasteless gas that is commonly produced by the incomplete combustion of carbon-rich fuel [1]. Its remarkable affinity to hemoglobin (250 times more binding) displaces oxygen, reduces hemoglobin oxygen-carrying capacity and results in tissue hypoxia [1]. The latter event, coupled with a direct mitochondrial electron transfer dysfunction, culminates in a pathological, hypoxia-driven cascade of events responsible for the short-term and long-term complications of CO poisoning [1].

Symptomatology, ranging from headache and dizziness progressing to convulsions, coma or cardiopulmonary arrest and death, are directly related to the degree and severity of poisoning. Later complications in survivors include extrapyramidal dysfunction and neuropsychiatric and cognitive difficulties. Early suspicion and diagnosis, high-dose oxygen supplementation proceeding to hyperbaric therapy if indicated, control of seizures and cardiopulmonary and metabolic support are the mainstays of a successful treatment and good outcome [1,3].

CO poisoning requires prompt management once it is diagnosed. The clinical assessment of the patient should include proper airway management and ventilation in addition to maintenance of hemodynamic stability [1].

Administration of high-flow oxygen should be initiated by emergency personnel before arrival at the hospital. Normobaric oxygen should be continued until the COHb level is less than 5 percent [1]. However, some hyperbaric oxygen (HBO) therapy experts prefer to lower the COHb level to less than 2 percent because some patients can have a rebound shift of CO from soft tissues into the circulation.

Essential preliminary assessment parameters should include: duration and source of exposure, a complete neurological examination, measurement of arterial blood gases looking specifically for the COHb level (this is because pulse oximetry cannot differentiate between carboxyhemoglobin and oxyhemoglobin), and identification of significant metabolic acidosis [3].

The COHb level depends on many factors such as the duration of exposure, the alveolar ventilation and blood volume [4]. The atmospheric concentration of CO is 0.1 percent. Levels in non-smokers are between 3 percent to 5 percent, while smokers may have higher levels reaching 6 percent to 8 percent [5]. The clinical symptoms may not necessarily correlate with the COHb level. They are thought to be a product of an inflammatory process that is initiated by the CO poisoning.

CO elimination is related to the fraction of inspired oxygen. The half-life of COHb is 250 minutes if the patient is breathing room air, 90 minutes if the patient is on 100 percent oxygen 200kPa and 35 minutes if on 100 percent oxygen 300kPa [6].

CT or MRI studies may be used to assess the presence of an acute ischemic brain injury if performed six hours or more following a significant CO exposure. Acutely, the commonest finding on neuroimaging is cerebral edema, while post-acute stage MRI T2-weighted imaging may show hyperintensities in the basal ganglia and atrophy in the hippocampi [7,8]. Basal ganglia lesions were found in 4 percent to 88 percent of patients in a prospective study of 73 patients by Hopkins *et al*. commonly of the globus pallidus. These are reported at one month and up to five years after CO poisoning [9].

In addition to cardiac toxicity, CO poisoning has been associated with several serious neurological complications such as amnesia, encephalopathy, dysarthria, brain hemorrhage, supranuclear gaze palsy, peripheral neuropathy and parkinsonism [10].

To date, only one case of non-convulsive status epilepticus secondary to acute CO poisoning has been reported in the English literature. The patient in that case was treated by HBO therapy but unfortunately the patient died of sepsis with multi-organ failure [11]. Our patient had severe CO poisoning manifested by coma, status epilepticus and cardiac arrest. Her COHb level was 21.4 percent, which was obtained two hours later after removal of our patient from the source of the exposure; therefore, the real COHb level during exposure was much higher considering the fast elimination of CO gas. Her refractory seizures, likely related to global cerebral hypoxia secondary to CO poisoning, abated after adding intravenous thiopental therapy to phenytoin and midazolam regiment. Hyperbaric oxygen was not utilized in our patient’s case as it was not available in our institution and our patient’s condition was initially too unstable for transfer to another facility.

Hyperbaric oxygen therapy is defined as the breathing of 100 percent oxygen within hyperbaric chambers at 1.4 times atmospheric pressure [12].

Hyperbaric oxygen has a neuroprotective effect as it leads to cerebral vasoconstriction, thereby reducing cerebral edema [13]. Earlier use of HBO contributes to a better outcome [14]. It is specifically indicated for victims of carbon monoxide poisoning presenting with coma, seizures or cardiac arrhythmia [15]. HBO was clearly indicated in our patient. However she made a full recovery with normobaric oxygen (NBO) treatment delivered by conventional mechanical ventilation.

HBO versus NBO treatment has been studied in human patients in only few randomized clinical trials. Most of them included acute presentations of non-comatose patients. No difference in the short-term outcome between HBO and NBO was found [16].

Scheinkestel and colleagues found HBO therapy did not benefit, and may have worsened, the outcome in the learning test at completion of treatment. Delayed neurological sequelae were restricted to HBO patients and no outcome measure was worse in the NBO group [17].

Weaver *et al*. performed a double-blinded, randomized trial of hyperbaric oxygen compared with normobaric oxygen therapy in acute CO poisoning. The trial was stopped prematurely after an interim analysis showed that patients treated with hyperbaric oxygen had less frequent cognitive dysfunction compared with patients treated with normoxia which was persistent at 12 months of follow-up [18].

A 2011 Cochrane meta-analysis review of all randomized trials did not establish whether administration of HBO to victims of CO poisoning reduced the incidence of neurological late sequelae [19].

In one study, HBO was found to be most appropriate and beneficial for patients with acute CO poisoning who are 36 years or older or have an exposure interval of more than 24 hours. Loss of consciousness and higher COHb levels warranted HBO in a subgroup analysis by randomized controlled trial. Of 163 patients who did not receive HBO, 42 percent had cognitive complications [20].

Narcotics such as fentanyl and morphine are considered for cases of severe traumatic brain injury as they provide analgesia, mild sedation and cough suppression, which might elevate the intra-cranial pressure (ICP) in intubated and mechanically ventilated patients. Sedation can potentiate the effect of analgesia because of the anxiolytic effect and prevention of elevation of ICP due to agitation, discomfort, cough, pain, facilitating nursing care and mechanical ventilation. Narcotics with sedative medications could decrease the oxygen consumption and carbon dioxide production as well, which affects the ICP [21].

Barbiturates are proven to be effective for status epilepticus treatment and decreasing ICP by reducing the cerebral metabolism and cerebral blood flow [21].

Hypotension has been associated with poor outcome post-traumatic brain injury, therefore adequate volume resuscitation by isotonic fluid to achieve euvolemia and initiating vasopressor infusion to maintain MAP ≥80mmHg may be helpful to maintain cerebral perfusion pressure within normal limits [21].

Hyperglycemia is an important insult to the brain due to stress after severe brain injury. It can lead to cerebral edema and poor neurological outcome [21].

Additionally, hypothermia was helpful in our patient because it is able to reduce the metabolic rate in brain cells, the oxygen demand and cerebral edema. It may also ameliorate toxic metabolites and free radical formation. The risks of hypothermia use include infection, coagulopathy and arrhythmias [21-23].

Despite the aggressive presentation in our patient’s case, with status epilepticus and cardiac arrest, she made a full recovery with normobaric oxygen therapy. Based on our experience, we believe that normobaric mechanical ventilation when accompanied by proper methods of central nervous system protection (hypothermia, normoglycemia, phenobarbital) can be an option for CO poisoning victims if hyperbaric oxygen therapy is not available.

## Conclusions

In summary, we report a case of severe carbon monoxide poisoning presenting with status epilepticus and cardiac arrest. Our patient was treated with normobaric oxygen therapy and strict neuroprotection with a full functional recovery. The findings in the present case suggest that normobaric high concentration of oxygen coupled with neuroprotection may be a therapeutically adequate alternative to hyperbaric oxygen for the treatment of severe CO poisoning.

## Consent

Written informed consent was obtained from the patient for publication of this manuscript and any accompanying figures. A copy of the written consent is available for review by the Editor-in-Chief of this journal.

## Competing interests

The authors declare that they have no competing interests.

## Authors’ contributions

SA and IH: wrote and revised the manuscript. OD: managed our patient in the Emergency Room and Intensive Care Unit, and provided critical care perspectives in the Discussion. SK: was the neurologist following our patient regarding the anti-epileptic drugs and interpreting EEG findings, and reviewed the paper from neurology perspectives. YA: participated in the management of our patient’s condition, and made the final review of the case report. All authors read and approved the final manuscript.

## References

[B1] Ernst A Zibrak JD Carbon monoxide poisoning N Engl J Med 1998 339 1603 1608 10.1056/NEJM199811263392206 9828249

[B2] Arabi YM Haddad S Tamim HM Al-Dawood A Al-Qahtani S Ferayan A Al-Abdulmughni I Al-Oweis J Rugaan A Mortality reduction after implementing a clinical practice guidelines-based management protocol for severe traumatic brain injury J Crit Care 2010 25 190 195 10.1016/j.jcrc.2009.05.004 19592201

[B3] Touger M Gallagher EJ Tyrell J Relationship between venous and arterial carboxyhemoglobin levels in patients with suspected carbon monoxide poisoning Ann Emerg Med 1995 25 481 483 10.1016/S0196-0644(95)70262-8 7710152

[B4] Peterson JE Stewart RD Absorption and elimination of carbon monoxide by inactive young men Arch Environ Health 1970 21 165 171 5430002 10.1080/00039896.1970.10667215

[B5] Hee J Callais F Momas I Laurent AM Min S Molinier P Chastagnier M Claude JR Festv B Smokers’ behaviour and exposure according to cigarette yield and smoking experience Pharmacol Biochem Behav 1995 52 195 203 10.1016/0091-3057(95)00089-F 7501665

[B6] Jay GD McKindley DS Alterations in pharmacokinetics of carboxyhemoglobin produced by oxygen under pressure Undersea Hyperb Med 1997 24 165 173 9308139

[B7] Pracyk JB Stolp BW Fife CE Gray L Piantadosi CA Brain computerized tomography after hyperbaric oxygen therapy for carbon monoxide poisoning Undersea Hyperb Med 1995 22 1 7 7742705

[B8] Gale SD Hopkins RO Weaver LK Bigler ED Booth EJ Blatter DD MRI, quantitative MRI, SPECT, and neuropsychological findings following carbon monoxide poisoning Brain Inj 1999 13 229 243 10.1080/026990599121601 10230524

[B9] Hopkins RO Fearing MA Weaver LK Foley JF Basal ganglia lesions following carbon monoxide poisoning Brain Inj 2006 20 273 281 10.1080/02699050500488181 16537269

[B10] Bennetto L Powter L Scolding NJ Accidental carbon monoxide poisoning presenting without a history of exposure: a case report J Med Case Rep 2008 22 2 118 10.1186/1752-1947-2-118 PMC2390579 18430228

[B11] Brown KI Wilson RF White MT Carbon monoxide induced status epilepticus in an adult J Burn Care Res 2007 28 533 536 10.1097/BCR.0B013E318053DA82 17438487

[B12] Gesell LB (Ed): Hyperbaric Oxygen 2009: Indications and Results: The Hyperbaric Oxygen Therapy Committee Report 2008 Durham, NC: Undersea and Hyperbaric Medical Society

[B13] Mori T Nagai K Carbon-monoxide poisoning presenting as an isolated afebrile seizure Pediatr Neurol 2000 22 330 331 10.1016/S0887-8994(99)00148-4 10788755

[B14] Bentur Y Hyperbaric oxygen for carbon monoxide poisoning Toxicol Rev 2005 24 153 154 10.2165/00139709-200524030-00004 16390213

[B15] Myers RAM Thom SR Kindwall EP Carbon monoxide and cyanide poisoning Hyperbaric Medicine Practice Myers RAM, Thom SR 1994 Flagstaff, AZ: Best Publishing 357

[B16] Raphael JD Elkharrat D Jars-Guincestre MC Trial of normobaric and hyperbaric oxygen for acute carbon monoxide intoxication Lancet 1989 2 414 419 2569600 10.1016/s0140-6736(89)90592-8

[B17] Scheinkestel CD Bailey M Myles PS Jones K Cooper DJ Millar IL Tuxen DV Hyperbaric or normobaric oxygen for acute carbon monoxide poisoning: a randomized, controlled clinical trial Med J Aust 1999 170 203 210 10092916 10.5694/j.1326-5377.1999.tb140318.x

[B18] Weaver LK Hopkins RO Chan KJ Churchill S Elliott CG Clemmer TP Orme JF Jr Thomas FO Morris AH Hyperbaric oxygen for acute carbon monoxide poisoning New Engl J Med 2002 347 1057 1067 10.1056/NEJMoa013121 12362006

[B19] Buckley NA Juurlink DN Isbister G Bennett MH Lavonas EJ Hyperbaric oxygen for carbon monoxide poisoning Cochrane Database Syst Rev 2011 13 CD002041 10.1002/14651858.CD002041.pub3 PMC7066484 21491385

[B20] Weaver LK Valentine KJ Hopkins RO Carbon monoxide poisoning: risk factors for cognitive sequelae and the role of hyperbaric oxygen Am J Respir Crit Care Med 2007 176 491 497 10.1164/rccm.200701-026OC 17496229

[B21] Haddad SH Arabi YM Critical care management of severe traumatic brain injury in adults Scand J Trauma Resusc Emerg Med 2012 3 12 10.1186/1757-7241-20-12 PMC3298793 22304785

[B22] Busl KM Greer DM Hypoxic-ischemic brain injury: pathophysiology, neuropathology and mechanisms NeuroRehabilitation 2010 26 5 13 20130351 10.3233/NRE-2010-0531

[B23] Kamijo Soma K Severe carbon monoxide poisoning complicated by hypothermia: a case report Am J Emerg Med 2011 29 357. e5-7 20674229 10.1016/j.ajem.2010.04.011

